# Cerebral Venous Sinus Thrombosis and Hydrocephalus in a Vegan Secondary to Acquired Hyperhomocystinaemia

**DOI:** 10.1155/2019/1468704

**Published:** 2019-12-10

**Authors:** W. D. D. Priyankara, A. V. I. Chandimal, F. G. Sivagnanam, E. M. Manoj

**Affiliations:** ^1^Consultant Intensivist, National Hospital of Sri Lanka, Colombo, Sri Lanka; ^2^Registrar in Medicine, National Hospital of Sri Lanka, Colombo, Sri Lanka; ^3^Consultant Physician, National Hospital of Sri Lanka, Colombo, Sri Lanka

## Abstract

Cerebral venous sinus thrombosis associated with acute hydrocephalus and periventricular leukoencephalopathy is a challenging combination, in a critically ill with deteriorating neurology. We report a case of a young man with acute onset neuropsychiatric manifestations, admitted to the intensive care unit. He was found to have widespread cerebral venous sinus thrombosis, hyperintensities in basal ganglia, and acute hydrocephalus in magnetic resonance imaging, necessitating cerebrospinal fluid diversion, by way of an external ventricular drain and therapeutic anticoagulation. He had otherwise normal routine biochemistry, except for macrocytosis, which prompted us to suspect acquired hyperhomocysteinemia secondary to cobalamin and folate deficiency, in the background of him being a vegan. Replacement of vitamin B_12_, folic acid, pyridoxine along with anticoagulation and control of intracranial pressure with external ventricular drain lead to dramatic improvement of his neurology. Therefore, high index of suspicion is crucial for a better outcome in otherwise irreversible neurological damage in acquired hyperhomocystinaemia.

## 1. Introduction

Cerebral venous sinus thrombosis (CVT) is an uncommon condition that poses diagnostic challenge to clinicians due to myriad causes and presentations [1, 2]. Furthermore, hydrocephalus is rare in CVT [3, 4]. Elevated homocysteine levels in plasma increase the risk of arterial as well as venous thrombosis [5]. Cobalamin (vitamin B_12_) and/or folate deficiency are recognized causes of hyperhomocystinaemia [6, 7]. We report a case of encephalopathy, CVT, and hydrocephalus secondary to acquired hyperhomocystinaemia due to cobalamin and folate deficiency in a vegan.

## 2. Case Report

A 24-year-old male admitted to the hospital with severe headache followed by altered sensorium and involuntary movements of the face and right upper limb for 2 days. He was drowsy with Glasgow Coma Scale (GCS) of 12/15 and generalized tonicity. His optic fundus showed papilledema with left hemiplegia. Further clinical examination was unremarkable with heart rate of 70 bpm, blood pressure 138/68 mmHg, respiratory rate 28 per minute, and oxygen saturation of 96% on air. He was initially treated as for meningoencephalitis with intravenous cefotaxime and acyclovir. His computed tomography (CT) scan of brain has shown hypodensities in basal ganglia and temporal lobes.

**Figure 1:**
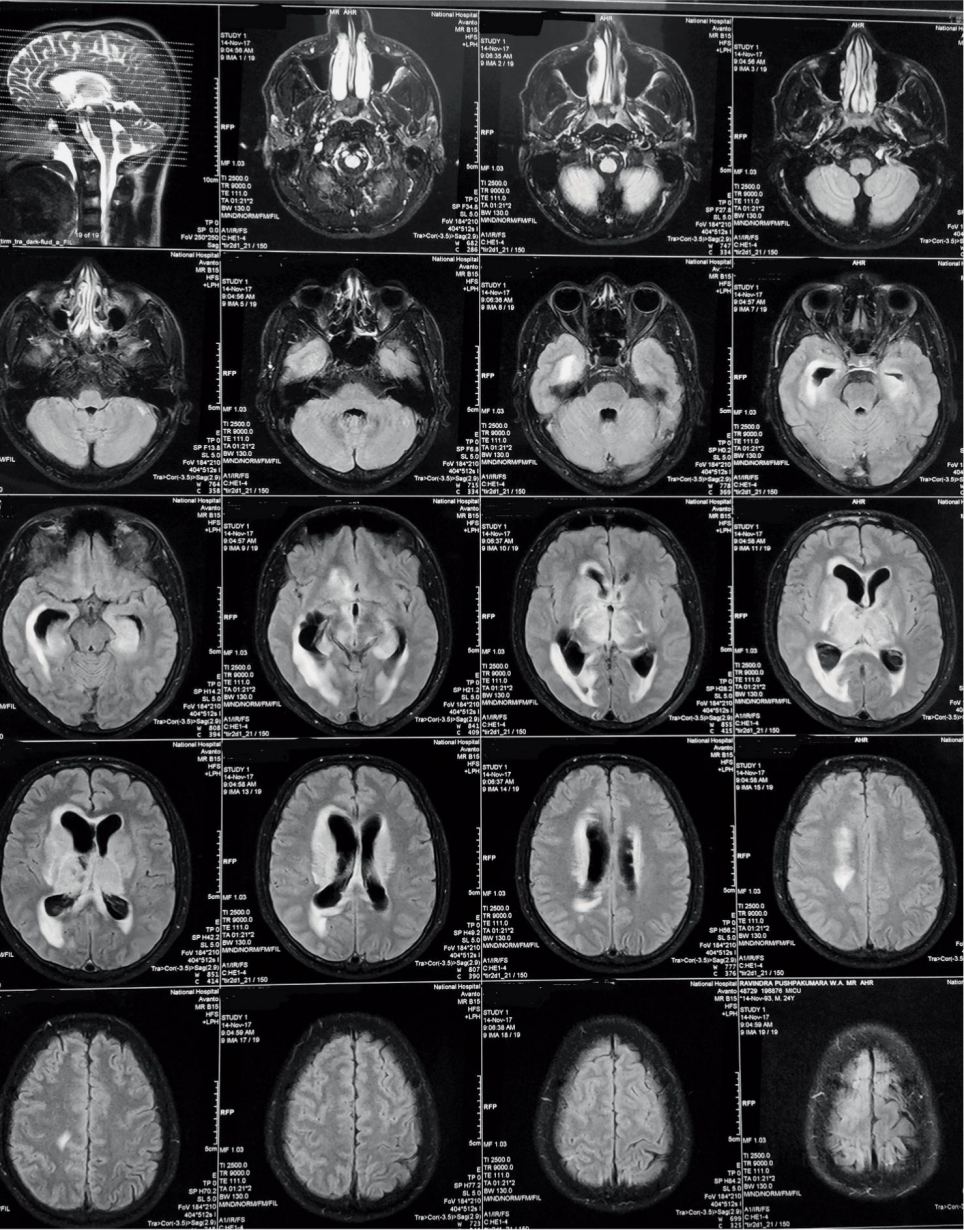
MRI showing hydrocephalus, periventricular oedema and leukoaraiosis.

**Figure 2:**
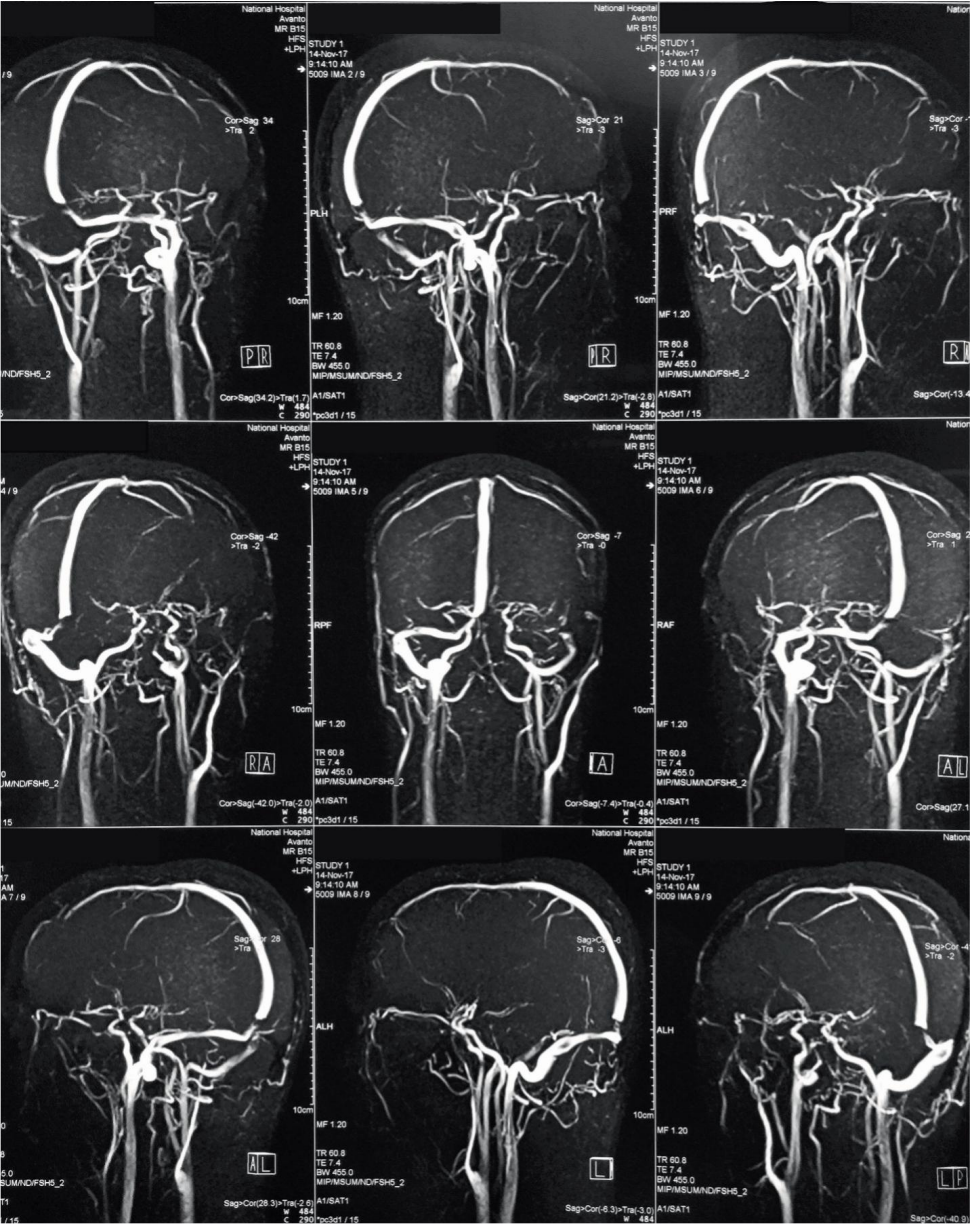
MRV showing thrombosis of the straight sinus, posterior part of superior sagittal sinus and right transverse sinus.

Over the next 24 hours, his condition further deteriorated with worsening respiratory distress and drop in GCS to 8/15 associated with bradycardia and hypertension (Cushing's reflex) suggestive of rapidly rising intracranial pressure. He was transferred to the intensive care unit (ICU) and started on invasive ventilation targeting brain protective measures. Magnetic resonance imaging (MRI) of the brain with arteriography and venography, revealed thrombosis of the straight, superior sagittal and right transverse sinuses associated with hemorrhagic infarcts in bi-lateral basal ganglia, thalami, and diencephalon with acute hydrocephalus and periventricular leukoencephalopathy (Figures 1 and 2). Thereafter, patient underwent urgent insertion of an external ventricular drain (EVD) followed by therapeutic anticoagulation with subcutaneous low molecular weight heparin. His cerebrospinal fluid (CSF) analysis was unremarkable except for raised proteins of 190 mg/dl. Subsequent exploration of a cause for his clinical picture, including coagulation profile, antinuclear antibodies (ANA), double stranded DNA (dS-DNA), antineutrophil cytoplasmic antibodies (ANCA), anti-beta 2 glycoprotein, anticardiolipin antibodies, NMDAR antibodies, and JACK2 mutation, was unremarkable. Blood film showed macrocytosis with raised red cell mean corpuscular volume of 107 fl/r. His serum and red cell folate and serum B_12_ levels, were low, leading us to suspect acquired hyperhomocystinaemia (H-Hcy). His serum homocysteine levels were more than 50 *µ*mol/l (5.4–16.1 *µ*mol/l). Then, he was started on vitamin supplements; B_12_ 1,000 mcg per day for two weeks, folic acid 5 mg daily and pyridoxine 25 mg daily with a dramatic improvement of his neurology, managing to extubate on day 6 and remove EVD successfully on day 7 of ICU admission. Patient was discharged after 15 days of hospital stay without any residual neurology, on warfarin (aiming at INR 2–2.5) and vitamin supplements. On follow up at 12 weeks, his serum homocysteine level has normalized and vitB_12_ and folate levels were normal. Warfarin was stopped at 3 months.

## 3. Discussion

CVT accounts for about 0.5–2% of all stroke cases in adults and carries a high morbidity and mortality rate [1, 2]. Thrombosis leads to impaired venous out flow and spinal fluid drainage resulting in increased intracranial pressures (ICP). However, reported incidence of hydrocephalus is rare due to CVT [3, 4]. A study done by Susanna Zuurbier et al., on patients with CVT demonstrated that hydrocephalus was mainly seen in patients with deep cerebral venous thrombosis and oedema of the basal ganglia and thalami and not due to the direct effect of venous thrombosis [8]. Authors assumed that lesions in bilateral basal ganglia region appears to be compressing 3^rd^ ventricle and foramen of Monro causing acute hydrocephalus which is a marker of severity of CVT. Hydrocephalus in our patient could be explained with the similar mechanism with secondary oedema of basal ganglia and thalami. Furthermore, bilateral symmetrical hyperintense signals in the basal ganglia, which is a rare manifestation of encephalopathy secondary to B12 deficiency has been reported [9, 10].

Inherited factor V Leiden and thrombin genetic mutations and antiphospholipid syndrome are well known to cause CVT [11–13]. Even though H-Hcy is associated with deep vein thrombosis, the association of CVT due to gene mutations in methylene tetrahydrofolate reductase (MTHFR) is controversial [7, 14]. Deficiency of vitamin B_12_, folate can raise the blood homocysteine levels [6]. Study done by Martinelli et al. showed that H-Hcy increases the risk of CVT by four fold [5]. Furthermore, a study carried out by Carlos Cantu in Mexico showed H-Hcy and folate deficiency was associated with increased risk of CVT [7].

Cobalamin is vital to the function of neurons and brain aging status. Thus, its deficiency may lead to not only brain dysfunction, but structural lesions, causing neuropsychiatric manifestation explained by several mechanisms. Radiologic manifestations of cobalamin deficiency or H-Hcy include leukoaraiosis, which was evident in our patient as periventricular leukoencephalopathy in the MRI.

Our patient was a strict vegan and had folate and vitamin B_12_ deficiency. We were not able to perform MTHFR gene mutations due to unavailability. Although most patients respond well to cobalamin treatment, residual neurology persists in most. Thus, treating deficiencies in the early stages is commonly accepted to yields better results, as structural and irreversible changes in the brain may occur if left untreated. Vitamin B_12_ status has been associated with the severity of periventricular white-matter lesions, and as a consequence, early detection and treatment of vitamin B_12_ deficiency [10]. Our patient's homocysteine levels had come to normal levels in 12 weeks with vitamin supplementation suggesting H-Hcy was purely due to folate and vitamin B_12_ deficiency.

## 4. Conclusion

Hyperhomocysteinemia induced venous sinus thrombosis and subsequent hydrocephalus is rare. Our case highlights that a high degree of suspicion is needed to diagnose hyperhomocysteinemia as a cause of CVT. Early detection and treatment of CVT is important to prevent structural and irreversible damage in affected individuals.
